# Hunger, satiety, and eating behavior after craniopharyngioma – narrative review on current management and future therapeutic perspectives

**DOI:** 10.3389/fendo.2026.1895026

**Published:** 2026-08-06

**Authors:** Hermann L. Müller

**Affiliations:** Department of Pediatrics and Pediatric Hematology/Oncology, University Children’s Hospital, Carl von Ossietzky Universität, Klinikum Oldenburg AöR, Oldenburg, Germany

**Keywords:** craniopharyngioma, eating behavior, hunger, hypothalamus, obesity, quality of life, satiety, setmelanotide

## Abstract

Craniopharyngiomas are low-grade, intracranial tumors frequently associated with dysfunction of the hypothalamic–pituitary axes and the development of obesity. This narrative review aimed to evaluate the potential contribution of eating behaviors, while also identifying perspectives for future therapeutic intervention. Searches were performed across five bibliographic databases. All peer-reviewed published studies involving childhood-onset or adult-onset craniopharyngioma populations were considered eligible when they examined eating behavior in relation to pharmacological or surgical interventions, neuroimaging, endocrine responses, energy expenditure, sleep, and/or neuropsychological outcomes. The available evidence regarding eating behavior in individuals with craniopharyngioma was limited, with only 10 of the 82 included studies employing validated measures of eating behavior. Eating behavior was assessed as a primary outcome in seven studies, as a secondary outcome in twelve studies, and discussed without formal measurement in thirty-one studies. Only one randomized controlled trial with the melanocortin-4 receptor (MC4R) agonist setmelanotide was identified that successfully targeted eating behavior (hyperphagia) modification and achieved significant weight loss as an intervention for craniopharyngioma-related obesity. Greater insight into eating behavior in this population, supported by long-term studies using validated assessment tools, is required to clarify the mechanisms underlying craniopharyngioma-associated obesity and confirm effectiveness and tolerability of medical treatment with MC4R-agonists.

## 1 Introduction

Craniopharyngiomas are embryonal, tumorous malformations that develop along the craniopharyngeal duct (1–4). The incidence ranges from 0.5 to 2 cases per million individuals annually (5, 6), with 30–50% of cases arising in pediatric populations (7). These tumors are histologically classified as low-grade (WHO I°) (8), and long-term survival rates are high (approximately 80% survival at 30 years (9, 10)). However, due to their anatomical location, they are associated with considerable clinical burden and substantial morbidity (11, 12), including visual (13, 14), endocrine (15–17), cardiovascular (18), and neurological complications (19) (20), as well as impairments in cognition and learning (21). Hypothalamic syndrome is an umbrella term describing a constellation of the above-mentioned clinical manifestations resulting from hypothalamic damage or dysfunction (22–25). Current clinical guidelines advocate for a more conservative therapeutic strategy (26–30), partly due to evidence indicating a substantial increase in the prevalence of severe obesity (22–62%) following radical neurosurgical intervention (1, 25, 31–35).

Hypothalamic obesity – as a part of hypothalamic syndrome - refers to excessive weight gain resulting from disruption of hypothalamic pathways that regulate satiety and energy homeostasis (36, 37). Damage to these neural circuits can significantly disturb energy balance and appetite regulation (22, 23, 38, 39). In the context of craniopharyngioma, the severity of obesity has been linked to the degree of hypothalamic injury (40–48). However, given the rarity and clinical complexity of these tumors (31), reliable definitions of diagnostic criteria (28, 49, 50) and effective therapeutic options for hypothalamic obesity remain limited, despite its profound impact on quality of life (1, 27, 51–53).

Multiple physiological and neurological factors associated with hypothalamic dysfunction in hypothalamic syndrome such as pituitary deficits, as well as broader neuro disability may contribute to altered eating behavior and subsequent weight gain. Existing evidence indicates that individuals with craniopharyngioma exhibit alterations in enteroendocrine and adipose-derived hormones involved in appetite regulation, nutrient processing, and insulin secretion (2, 54–58). For instance, elevated circulating levels of insulin and leptin have been observed compared to individuals with non-craniopharyngioma-related obesity (59). Conversely, compared to patients with non-functioning pituitary adenomas, those with adult-onset craniopharyngioma demonstrate reduced fasting ghrelin levels and attenuated postprandial ghrelin suppression (60), alongside decreased fasting oxytocin concentrations in cases with anterior hypothalamic damage (61). In addition, reductions in physical activity and resting energy expenditure have been consistently reported (62–65), which may further contribute to weight gain (64). Sleep disturbances, including excessive daytime sleepiness and impaired nocturnal sleep, are also prevalent and may result from tumor-related disruption of circadian regulation (66–72). Such disturbances may indirectly influence eating behavior (73). Neuropsychological and neuroimaging research has further highlighted alterations in brain function associated with craniopharyngioma. Studies have demonstrated abnormal neural responses to food-related stimuli (74, 75), as well as deficits in memory retrieval linked to prefrontal cortex dysfunction (76). Structural changes, including alterations in the hippocampus, have also been reported (77). These findings align with broader evidence of cognitive (21) and behavioral (78) impairments in this population.

Collectively, these interacting factors suggest that the mechanisms underlying eating behavior and obesity in individuals with craniopharyngioma are complex and remain incompletely understood. To address this gap, this narrative review summarizes existing evidence on eating behavior of craniopharyngioma patients (38, 79, 80) and discusses future research directions and intervention strategies.

## 2 Methods

This narrative review aims to assess the occurrence and severity of eating behavior disorders such hyperphagia in CP patients, with and without hypothalamic obesity, and to analyze the symptoms of eating disorders.

### 2.1 Search strategy

The narrative review is based on a literature search of MEDLINE, Excerpta Medica Database, PsycINFO, Web of Science, and Scopus for initial identification of publications. The articles were identified using the following keywords: a) “craniopharyngioma” and b) “eating behavior,” “feeding behavior,” “appetite,” “food intake,” “hyperphagia,” “hunger,” “satiety,” “overeating,” “emotional eating,” “restrained eating,” “binge-eating-disorder,” “disordered eating,” “hypothalamic syndrome,” or “hypothalamic obesity.” Studies examining related factors were also included when these variables were shown to influence eating behavior. Such factors comprised pharmacological and surgical interventions, imaging findings, endocrine responses, energy expenditure, sleep, and neuropsychological outcomes. Selected English language papers published before April 2026 were included in our review. Only studies published in peer-reviewed journals were considered.

### 2.2 Definitions, inclusion and exclusion criteria

**Figure 1:**
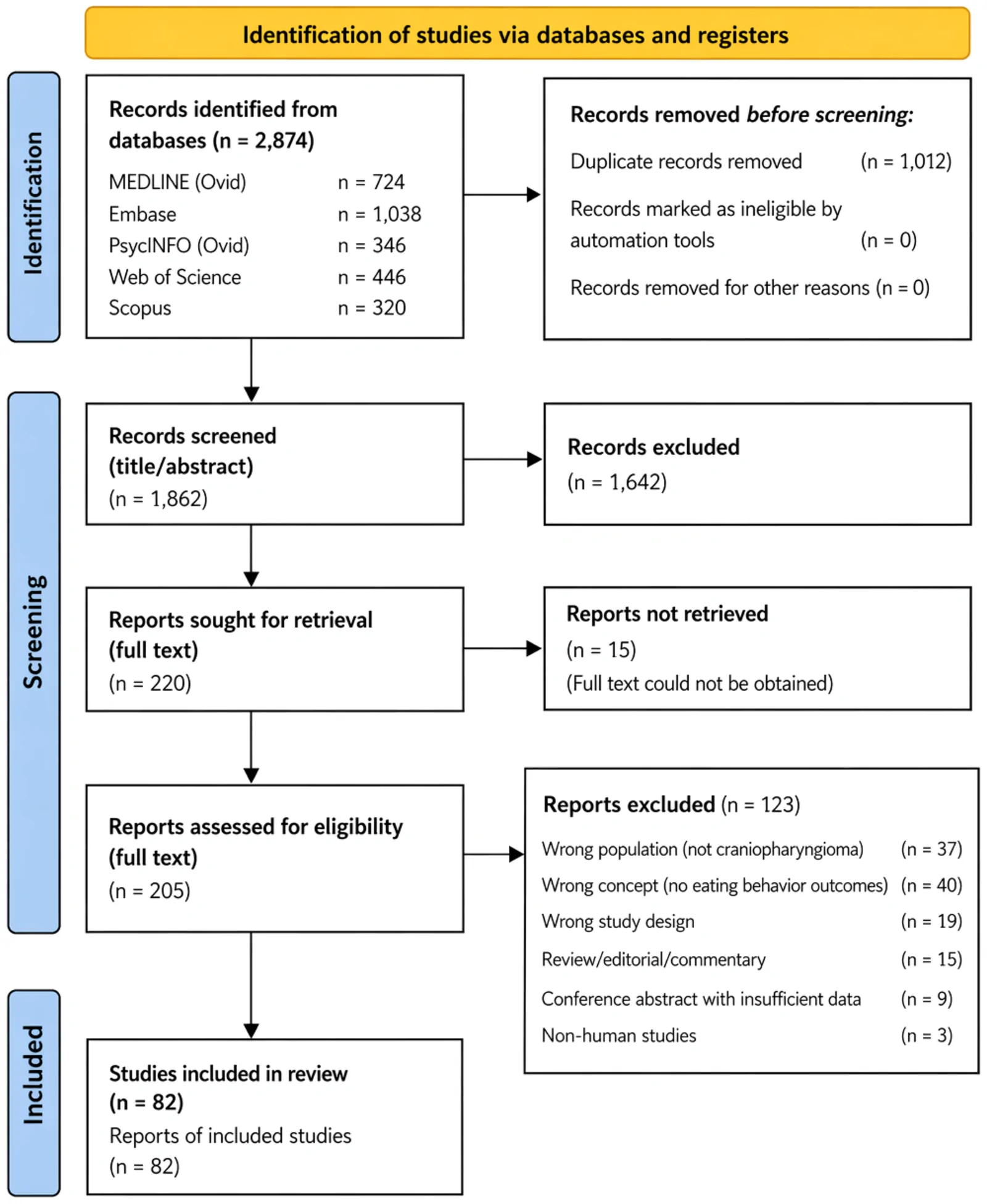
PRISMA 2020 flow diagram for studies of eating behavior in craniopharyngioma patients (search up to 31 April 2026).

The inclusion criteria, based on the PICOS approach, were as follows: participants (P): patients diagnosed with craniopharyngioma (both childhood-onset or adult-onset craniopharyngioma); intervention (I): no restrictions; comparison (C): no restrictions; outcomes (O): parameters related with eating behavior; study design (S): original studies. Exclusion criteria included: 1) animal studies or *in vitro* research; 2) studies involving participants with craniopharyngioma with additional central nervous system complications or patients with alternative causes of hypothalamic obesity; 3) reviews, case report, thesis, and conference proceedings. No limitations were imposed on language, or country of origin in order to capture the full extent of the available literature. In cases where multiple studies were published based on the same databases, only the study with the largest sample size was included in the analysis. Eighty-two publications fulfilled the above-mentioned criteria. (**Figure 1**).

The data extracted from all studies in line with our research objectives included participants characteristics (i.e., age of the subjects, age of tumor onset, clinical characteristics), procedures used to evaluate eating disorders (subjective and/or objective investigations), type of eating disorder, any additional examinations, treatment and efficacy, if available.

### 2.3 Study selection

All records retrieved from the literature search were imported into EndNote v.21 (Clarivate) for reference management and initial duplicate removal. The remaining records were subsequently transferred to the open-source systematic review platform Rayyan for additional deduplication and study screening. Titles and abstracts were independently screened against the predefined eligibility criteria. Full-text articles of potentially eligible studies were retrieved and assessed for inclusion by the primary reviewer (HLM). Reasons for exclusion at the full-text stage were documented. Any uncertainties or disagreements regarding study eligibility were resolved through assessment by a second independent reviewer.

### 2.4 Data extraction

Data from all eligible studies were extracted by the primary reviewer using a standardized data extraction framework. Extracted variables included publication characteristics, participant characteristics, study objectives, design, setting, outcome measures, principal findings, and authors’ recommendations. Any discrepancies identified during the extraction process were reviewed and resolved by a second independent reviewer. Evidence-quality assessment of included studies was not part of this narrative review.

## 3 Results

### 3.1 Type of studies addressing eating behavior in patients with craniopharyngioma

A total of 2,874 records related to eating behavior in patients with craniopharyngioma were identified across all data sources. Following the screening process, 2,792 records were excluded, resulting in the inclusion of 82 articles in the final analysis. A flow diagram summarizing study selection, categorization of records, and reasons for exclusion is presented in **Figure 1**. Overall, the available evidence regarding eating behavior in patients with craniopharyngioma remains limited, with relatively few studies providing high-quality data. Only 25% (20/82) of the included publications utilized validated questionnaires to assess eating behavior (**Table 1A**). The characteristics and findings of the included studies are summarized in **Tables 1A, B**. Approximately half of all publications (40/82) consisted of case reports or case series, whereas only 10% (8/82) were randomized controlled trials (RCTs), reflecting the generally low level of evidence in this field. In addition, 34% (27/82) of the studies specifically investigated the relationship between pharmacological interventions and eating behavior. Energy/caloric intake was quantified in 8 studies; by nutritional diary in 5/8 studies (62, 63, 65, 81, 82), by data of patient records in 1/8 studies (83), by dietitian-led food-recall in 1/8 studies (84), and by patient subjective assessment of prospective food consumption via visual analogic scale (VAS) in 1/8 studies (85) (**Tables 1A, B**).

**Table 1A:** Details of 20 studies (cohorts >1 case; listed in chronological order of publication) analyzing eating behavior after craniopharyngioma by standardized and validated instruments (157–175).

Study type	Cohorts/age at study	Initial parameters	Intervention/diagnostic instruments	EB tests	Findings/outcome	Authors/year/(ref.)
CCS	Cases; n=14 hypoth. tumors. Age 48.4 yrs; Controls; n=15 matched for age, BMI, % body fat, 48 yrs	Cases: median BMI 37.8 kg/m^2^; Controls: median BMI 37.5 kg/m^2^	None/test meal, Ghrelin, PYY,	TFEQ	HL: impaired satiety; not related to ghrelin and PYY changes after test meal	Daousi et al., 2005 (141)
CCS	Cases; n=42 childhood; age 28 yrs; Controls; n=42 matched for: age, sex, and residence, age NR	Cases: median BMI 30 Controls: median BMI 23	None/EE, energy intake (ND)	TFEQ	HL reduced sensitivity for leptin, insulin, ghrelin, and EE and physical activity	Holmer et al., 2010 (63)
CCS	Cases; n=31 with CP; 26 PA; Controls; n=114 healthy, matched for: gender and age. Mixed cohort of child-/adult onset	Cases: mean BMI 31.1 Controls: mean BMI 25.6	None/BDI, STAI-X, SF-36, QoL-AGHDA, ESS, EPQ-RK,	FEV, EDE-Q	higher hunger perception, negative mood alterations and anxiety-related traits	Roemmler-Zehrer et al, 2015 (89)
CCS	Cases; n=101 childhood, age 24.7 yrs. Controls; n=85 healthy, mat-ched for age, gender and BMI	NR, BMI range: SDS ≥+8 to BMI ≤+3	None/eating behavior (record data)	IEG, ESI	HO is associated with EB disorders in CP	Hoffmann et al., 2015 (83)
CCS	Cases: n=34 childhood-onset, age 20 yrs. Controls; n=73 healthy, age 39 yrs, matched for BMI	Cases: BMISDS median 4.3. Controls: BMISDS median 0.4	None/oxytocin in saliva	IEG, ESI	Association of EB with HL	Daubenbüchel et al., 2019 (86)
CCS	Cases; n=8, 10.5 yrs; Controls; n=8 matched: BMI, age, puberty, sex	Cases: BMI SDS 2.12; Controls BMI SDS 2.23	None/OGTT, insulin, GLP-1	HQCT DYQ	HO with impaired satiety, hyperphagia	Chai-Udom et al., 2020 (140)
RCT	n=22; 14 Tesomet, (6 CP); 8 placebo (4 CP)	at start: Tesomet BMI 37.3 ± 5.6, Placebo BMI 37.8 ± 5.8	tesofensin 0.5/met-oprolol 50, 24 wk	CSAS, VAS	mean weight loss of ~7.4 kg (6.6%) over 24 wk	Huynh et al., 2022 (155)
RCT	n=42: exenatide n=23: placebo n=19; median age 16 yrs.	Exenatide: mean BMI 38 Placebo: mean BMI 38	Exenatide ExQW 36 wk/BC, EE	CEBQ	decreased food intake and EE, not related with BC	Shoemaker et al., 2022 (107)
CCS	Cases; n=29, age 48 yrs, adult; controls n=31, age 49 yrs. PA	Cases: BMI postop 27 Controls/PA: BMI postop 25	None/fMRI food cue reactivity, PSQI	DEBQ, YFAS	HL causes weight gain by altering satiety/EB	Lee et al., 2023 (150)
CSS	Cases: n=54; HO with surgery: n=8, mean age 33.6 yrs; HO con-trols: n=10, 37.3 yrs. CO surgery: n=12, 38.8 yrs. CO controls: n=12, 33.9 yrs. Lean contr.: n=12, 29 yrs	Mean BMI: HO surgery: 48.7; HO control: 44.7; CO surgery: 38.5; CO control: 47.2; Lean control: 23	Bariatric surgery/fMRI, sweet preference test	FCQT, FEV, FEVII	fMRI/HO: increased acti-vation insula/cerebellum to viewing high-caloric foods. Bariatric surgery effective only without HL.	Dischinger et al., 2023 (90)
RCT	n=13, median age 15.3 yrs	BMI 33.1, median BMI z score (<18 yrs): +2.21	Oxytocin/QoL, test meal	DYQ, TFEQ	no significant impact of intranasal oxytocin on HO	McCormack et al., 2023 (105)
Case series	n=4 HO, 1 AO, 3 CO, age: 22, 44, 57, 69 yrs	Average weight 126.0 kg (78.8–172.0 kg)	semaglutide 1.7-2.4 mg/wk, 6 mo	TFEQ	Average weight loss 17% (11.3–22.4%)	Gjersdal et al., 2024 (110),
CCS	Cases: n=11; CO: age 14 yrs; Con trols; n=11; age 12 yrs, matched sex, pubertal stage age.	Cases: BMI SDS 1.4 Controls: BMI SDS -0.5	None/EE, OGTT, MRI, BC	CEBQAEBQ	association between BMI SDS/EB with HL	Hinton et al., 2024 (87)
P2OL	n=18; mean age 15.0 yrs (SD 5.3)	mean BMI 38.0 (SD 6.5)	setmelanotide 3·0 mg/d, 16 wk	DHS	45% reduced hunger score -15% BMI in 16/18 pts	Roth et al, 2024 (121)
RCT	n=40; n=20 randomized to exenatide, mean age 40.4 yrs n=20 randomized to placebo, mean age 40.4 yrs. Onset NR	Exenatide: BMI 30–34.9, n=12, 35–39.9 n=2, >40, n=6 Placebo: BMI 30–34.9 n=10, 35–39.9 n=2, >40, n=7	Exenatide, 26 wk/weight, caloric intake (ND), EE	TFEQ	Combined with intensive lifestyle interventions, exenatide was not superior to placebo to treat HO	Gatta-Cherifi et al., 2024 (81)
Case series	n=4 HO, 1 AO, 3 CO, age: 23, 45, 58, 70 yrs	Mean baseline BMI 48.0 ± 9.4 SD	semaglutide/1.7-2.4 mg/wk 24 mo DXA	TFEQ	-16% weight loss, impro-ved hunger/emotion score	Gjersdal et al., 2025 (111)
CCS	Cases: n=10, 12 yrs; Controls: n=9, 14 yrs, matched for: gender and age	Cases: mean BMI 28 Controls: mean BMI 32	None/Endocrino-logy+ energy intake (VAS) in CP/HL	VAS	HI and HO: reduced satiety persistence and earlier appetite rebound	Nogueira et al., 2026 (85)
Sur-vey	n=40 caregivers, childhood-onset CP	42.5% survivors with HO, 57.5% non-HO survivors	None	HQCT; FSZ	HO: low satiety, higher hunger/food preoccupation	Kayadjanian et al., 2026 (91)
CSS	n=21; 16 adult CP. median age 46.0 yrs (IQR 41.0–60.5)	Diagnostic grading of hypo-thalamic syndrome (49)	HL on MRI, eating behavior, weight	DYQ	Obesity: 47.6%, no hyper-phagia. 1 patient with HS	Tavares de Sil-va, 2026 (156)
RCT	Phase 3 RCT (2:1); n=120; 93 CP; Setmelanotide (SET), n=81, age: 19 yrs; placebo n=39; age: 21 yrs	BMI SDS: setmelanotide (SET): +3.72 SD; Placebo: +3.4 SD	setmelanotide (SET) 1.5-3.0 mg/d s.c., 52 wk	DHS	BMI: SET −16%; Placebo: +3%. Reduced DHS for SET compared to placebo (-2.73 *vs*. -1.45; p<0.05)	Miller et al., 2026 (123)

Abbreviations of instruments for analyses of eating behavior: AEBQ, Adult Eating Behaviour Questionnaire (160, 161); CEBQ, Child Eating Behaviour Questionnaire (158); CSAS, Composite Satiety Score (162); DEBQ, Dutch Eating Behavior Questionnaire (163); DHS, Daily Hunger Score (175); DYQ, Dykens Hyperphagia Questionnaire (159); EDE-Q, Eating Disorder Examination Questionnaire (164); FCQ-T, Food Cravings Questionnaire-Trait (165); FEV, Fragebogen zum Essverhalten (questionnaire for eating behavior) (174); FSZ, Food Safe Zone (166); HQCT, Hyperphagia-Questionnaire for Clinical Trials (167); IEG, Inventory for Eating Behaviour and Weight Problems (168) (173); ESI, Inventory for Eating Disorders (169); ND, nutritional diary; NQ, no questionnaire; SREB, Self-reported Eating Behavior; TFEQ, Three-Factor Eating Questionnaire (157, 170); VAS, Visual Analog Scale (171); YFAS, Yale Food Addiction Scale (172).

**Table 1B:** Details of 22 studies (cohorts >1 case; listed in chronological order of publication) analyzing eating behavior after craniopharyngioma by non-standardized and non-validated instruments.

Study type	Cohorts/age at study	Initial parameters	Intervention/diagnostic instruments	EB tests	Findings/ outcome	Authors/year/(ref.)
Case series	n=3, adult CP, severe HO post OP	extreme hyperphagia, abnor-mal food-seeking behavior	unsuccessful rehabilitation	NQ	poor outcome due to devi-ant behavior	Skorzewska et al., 1989 (182)
CCS	Cases; n=18 CP, onset NR. Control; n=16 PA, matched for: age, gender and yrs since surgery	Cases: mean BMI 31.5 PA Controls: BMI 30.9	None/non-vali-dated question-naire	NQ	Impaired experience of appetite and reduced con-trol of EB	Bellhouse et al., 2003 (88)
CCS	Cases: Group 1(G1): n=9, age 19.2 yrs; G2: n=27; Controls: 13.7 yrs. G1: n=11, 18 yrs, G2: n=15, 14 yrs	Cases: G1 mean BMI 2.9SD; G2: BMI 4.2SD; Controls: G1 BMI 2.7SD; G2: BMI 5.2SD	None/Energy intake (ND), actimetry	NQ	reduced physical activi-ty, normal caloric intake	Harz et al., 2003 (65)
RCT	n=18; 9 Octreotide (6 CP), 9 place-bo (7 CP); mean age 14.2 ± 0.7 yrs	Octreotide: 36.4 ± 2.4 Placebo: 36.2 ± 1.3	octreotide (5–15 ug/kg/d placebo, 6mo	NQ	Octreotide: QoL correla-ted with insulin response	Lustig et al., 2003 (143)
CCS	n=102, childhood-onset; age NR	With HL: n=42; w/o HL: n=60	None/functional capacity (FMH)	SREB	HO, HL, CP progression/relapse had QoL affects	Müller et al., 2005 (52)
CSS	n=27, age NR	NR	None/CBC	SREB	impaired body image due increased appetite/obesity	Ondruch et al., 2011 (148)
Case series	n=9 adults, 6 CP and other hypo-thalamic tumors	Mean BMI 37.6	1 liraglutide, 8 exenatide	SREB	8 patients: weight loss, 5/9 increased satiation	Zoicas et al., 2013 (109)
Case series	n=11; diencephalic syndrome in child CP; total cohort: 485 CP	21/485 BMI<-2SD preOP; 11/21 BMI<-2SD + HL	None/BMI, EB, energy intake	NQ, SREB	Cachexia changed to obe-sity during post OP FUP	Hoffmann et al., 2014 (94)
CCS	Cases; n=23, age 10 yrs; Control: n= 43 CO, age/sex-matched, 11 yr	Cases: mean BMI 29.4 Controls: mean BMI 29	None/BMI, EE, energy intake (ND)	NQ	Lower REE in CP regard-less of fat-free mass	Bomer et al., 2015 (62)
Single arm	n=8; CP n=6; mean age 27.5 yrs	Mean BMI 47.5 kg/m^2^	Exenatide/Food intake (recall), EE	NQ	Change in weight was not significant	Lomenick et al., 2016 (84)
CSS	n=39, 59% obese, median age 12.2 yrs,	Median BMI 25.6	None/BMI, EE, energy intake (ND)	NQ	Obese CP: Lower EE, similar energy intake.	Caminiti et al., 2017 (82)
RCS	n=5; child CP, mean age 15.4 (13–18) yrs	mean BMI SDS 3.39 ± 1.44	exenatide 2 mg/wk, 12 mo	SREB	Only 1 pat: weight loss (-5.4 kg, BMI SDS -0.33).	Van Schaik et al., 2020 (108)
RCT (107)	n=35; 20 ExQW, 15 placebo, age 10–25 yrs	Exenatide: mean BMI 35.2 Placebo: mean BMI 39.2	ExQW (2 mg/wk, placebo 36 wk	NQ	Exenatide+HL: %-body fat reduced but not BMI	Perez et al., 2021 (112)
RCT	n=41; 23 ExQW, 16.9 (4.3) yrs, 18 placebo; 16.9, (4.8) yrs	ExQW: BMI 35.8 (6.6) Placebo: BMI 39.2 (7.3)	ExQW 2 mg/wk; placebo 36 wk	NQ	no difference ExQW vs. placebo on %Δ BMI.	Roth et al., 2021 (113),
RCS	n=12; 8 CP, mean age 12.3 ± 4.0 yrs, dextroamphetamine treated	mean BMI SDS 3.32 ± 1.31	amphetamine max. 40 mg/d, 23 ± 12 mo	SREB	68% reduced hyperphagia, 10/12 pts: BMI reduction	Van Schaik et al., 2022 (183)
Sur-vey	n=82 caregivers, childhood-onset cases, mean age of cases 16.7 yrs	Health problems	None/PedsQL, ZBI	SREB	70% obesity, hyperphagia in HO/HL	Kayadjanian et al., 2023 (11)
RCS	n=106; median age 17.8 (14.1-22.0) yrs	BMI baseline: 0.4 (−0.4-1.4) SD, 5 yrs: 1.9 (0.8-3.3) SD	None/weight changes after CP	NQ	HO associated with female sex, HI, baseline BMI	Rovani et al., 2024 (184)
Obs. study	n=26, median age 52 (18–65) yrs	Pre OP median BMI 25 (20–38), therapy start: 38 (28–58)	semaglutide 1.6 (0.5-2.5) mg	NQ	15 (58%) lost >10% and 2 (8%) lost >20% of weight	Svendstrup et al., 2024 (114)
RCS	n=72; (26 pediatric, 46 adult), ini-tial endoscopic endonasal surgery	HL were independently asso-ciated with new/worsened HO	endoscopic endo-nasal surgery	NQ	worsened obesity in 17 patients (23.6%)	Honjo et al., 2025 (185)
PCS	n=47 childhood, age 9.7 yrs; PBT (≤ 22 yrs at the time of PBT)	Parent-proxy reports (PPR) Q on QoL using PedsQL	PBT in all patients/PEDsQoL	SREB	PPR on QoL was lower in patients with hyperphagia	Rose et al., 2025 (186)
CCS	n=99, adult CP	Preoperative HO 32% Postoperative HO 57%	risk factors of HO: pre-OP BMI, HL	NQ	Post OP weight gain (≥5%) in 71% patients	Klochkova et al., 2026 (187)
MCC	n=116; mean age 44.4 ± 13.5 yrs	mean BMI: 40.1 ± 8.6 kg/m^2^	semaglutide (1.2 mg/wk), 44 ± 39 mo	NQ	mean weight loss: 4.6% (± 12.5)	Lambert et al., 2026 (115)

EB, eating behavior; BC, body composition; BDI, Beck Depression Inventory; BMI, body mass index; CBC, child behavior checklist; CCS, case control study; CO, common obesity; CP, craniopharyngioma; CSR, Child-self reports; CSS, cross-sectional study; EE, energy expenditure; EPQ-RK, Eysenck Personality Questionnaire Revised; ESS, Epworth Sleepiness Scale; EuroQoL, Euro quality of life; ExQW, exenatide once-weekly extended-release; FUP, follow-up; HI, hypothalamic involvement; HL, hypothalamic lesion; HO, hypothalamic obesity; MCC, multicenter cohort; mo, months; NA, not applicable; NR, not recorded; OGTT, oral glucose tolerance test; PA, pituitary adenoma; P2OL, phase 2 open label study; PBT, proton beam therapy; PCS, prospective cohort study; PedsQL, Pediatric Quality of Life Inventory; PPR, Parent-proxy reports; QoL-AGHDA, quality of life in adult patients with growth hormone deficiency; RCS, retrospective cohort study; RCT, randomized-control study; SDS, standard deviation score; SF36, 36-item short form; STAI-X, State-Trait Anxiety Inventory; TPQ, Tridimensional Personality Questionnaire; wk, week; yrs, years; ZBI, Zarit Burden Interview.

### 3.2 Eating behavior in patients with craniopharyngioma

Alterations in eating behavior patterns have been documented among individuals with craniopharyngioma. Evidence has been derived from case control studies, case series, and an online survey (**Tables 1A, B**) (86, 87). Multiple studies have reported a higher prevalence of pathological eating behaviors in individuals with craniopharyngioma compared to matched controls without the condition (11, 86–90). Assessment using validated instruments has demonstrated several key findings in this population. These include heightened subjective hunger as measured by the Fragebogen zum Essverhalten (FEV) (90), increased hyperphagic tendencies identified via hyperphagia questionnaires (87, 89), reduced dietary restraint assessed using the Eating Disorder Examination Questionnaire (EDE-Q) (89), and behavioral patterns such as increased snacking frequency and greater parental pressure to eat during childhood, as measured by the Inventory for Eating Behavior and Weight Problems (IEG) (86). Additionally, patient-reported outcomes indicate active attempts at weight reduction (88). Caregiver-focused research suggests that behavioral manifestations associated with hyperphagia, rather than obesity itself, represent a management challenge (11, 91). Notably, only one study identified a direct association between hyperphagic behavior and increased body mass index (BMI) (87).

In contrast, two case–control studies (83, 86) reported that matched controls exhibited more pronounced pathological eating behaviors than individuals with craniopharyngioma when evaluated using the IEG questionnaire. Specifically, obese control participants (BMI >+3SD) demonstrated higher scores across domains such as hunger, food intake during special occasions, eating patterns, snacking behavior, and childhood feeding pressure (83, 86).

Findings using the Child Eating Behavior Questionnaire (CEBQ) were inconsistent (87). While individuals with craniopharyngioma showed increased engagement with food and greater interference of eating behaviors with daily functioning, obese controls exhibited more pronounced food avoidance traits, including slower eating, enhanced satiety responsiveness, and increased food selectivity (87). A longitudinal comparison of patient-reported hyperphagia before and after surgical intervention indicated an increase in reported hyperphagic symptoms following tumor resection, with 1/10 patients reporting symptoms preoperatively and 6/10 postoperatively (92).

Diencephalic syndrome, characterized by severe weight loss and cachexia, may also occur as a rare manifestation of hypothalamic dysregulation (93). Hoffmann et al. (94) investigated the incidence, clinical presentation before and after diagnosis, and outcomes of diencephalic syndrome in craniopharyngioma patients. At diagnosis, only 4.3% of 485 patients exhibited underweight (BMI <−2SD). Eating behavior could not be evaluated due to the retrospective design of the study and the fact that eating behavior was characterized as “reduced” based on individual assessment of the treating physician/caregiver/patient and not based on validated diagnostic instruments. During the first two years following diagnosis, BMI in patients with diencephalic syndrome and patients with normal weight tended to converge. Based on longitudinal patient data, Hoffmann et al. (94) concluded that the presence of diencephalic syndrome at diagnosis does not preclude subsequent weight gain associated with hypothalamic involvement in childhood-onset craniopharyngioma patients.

### 3.3 Interventions

#### 3.3.1 Lifestyle interventions

Lifestyle interventions reported to date have generally produced only short-term and transient reductions in BMI, suggesting that sustained benefits are unlikely without continued behavioral support and long-term coaching (23, 95). A key prerequisite for effective lifestyle management is a stable and supportive home environment, particularly given the combined challenges of persistent hyperphagia, pituitary dysfunction, and behavioral disturbances that may occur in this population (96). Evidence from a recent analysis of the German craniopharyngioma cohort (n=291) demonstrated that BMI at final follow-up was positively associated with both maternal and paternal BMI at diagnosis (97). These findings indicate that successful obesity management should extend beyond the individual patient and incorporate the wider family unit as well as caregivers (11).

#### 3.3.2 Pharmacological interventions

##### 3.3.2.1 Sibutramine

Two case studies investigating sibutramine reported reduced subjective hunger (98) and short-term improvements in BMI and energy levels, although weight regain occurred within one year (99).

##### 3.3.2.2 Stimulant medication

Evidence from one quasi-experimental study and case reports suggests that stimulant medications such as dextroamphetamine and mazindol may contribute to weight stabilization or reduction (100–102), along with decreased hunger perception (101, 103). However, long-term effectiveness appears to diminish over time (99).

##### 3.3.2.3 Naltrexone

A single case study demonstrated reduced subjective hyperphagia following naltrexone treatment, although a preference for high-carbohydrate foods persisted (104).

##### 3.3.2.4 Intranasal oxytocin

Three studies evaluated intranasal oxytocin (104–106). A pilot RCT found no measurable effects on eating behavior (105), whereas Mann et al. reported improvements in hyperphagia and food-related obsessive behaviors, with variable effects on body weight (69).

##### 3.3.2.5 Glucagon-like peptide-1 analogues

Eleven studies (81, 84, 107–115) have evaluated the effects of glucagon-like peptide-1 (GLP-1) analogues (4 semaglutide, 5 exenatide, 2 exenatide once-weekly extended-release), on eating behavior after craniopharyngioma (**Table 1**). In 4 of 11 studies standardized instruments were used to assess eating behavior. Increased eating slowness based on parental assessment of CEBQ (107), reduction of emotional and uncontrolled eating score (110, 111), and a reduction in hunger score (81) were observed. Weight changes under GLP-1 analogue therapy were variable, ranging from non-response (84, 112, 113, 115) to weight loss of 34 kg (109) and >10% in 58% of patients (114).

A randomized controlled trial (RCT) involving exenatide in 40 adults with craniopharyngioma demonstrated a reduction in hunger scores after 26 weeks compared to placebo; however, no significant effects were observed on disinhibition or dietary restraint using the Three-Factor Eating Questionnaire (TFEQ) (81). Conversely, another RCT reported improvement only in the “slowness of eating” domain of the CEBQ following exenatide treatment, without significant weight differences between treatment groups (107). Two quasi-experimental studies further examined exenatide (84, 107). One study in adults found reduced self-reported hunger without significant weight change (84), while another in younger individuals reported decreased energy intake and modest improvements in eating pace, although hyperphagia scores remained unchanged (107). Case reports and case series have generally indicated increased satiety (108, 109, 116, 117) and reduced hunger perception (108, 110, 111, 118–120), though these findings were not based on validated assessment tools. One patient, treated with liraglutide (109), reported less hunger and an increased feeling of satiety. After 8 months of therapy, the patient showed a reduction of BMI from 41.8 kg/m^2^ to 35.3 kg/m^2^.

##### 3.3.2.6 Melanocortin-4 receptor agonists

Setmelanotide, a melanocortin-4 receptor (MC4R) agonist, is the only pharmacological agent approved by the United States (U.S.) Food and Drug Administration (FDA) and the European Medicines Agency (EMA) for treatment of patients with hypothalamic obesity. Setmelanotide was assessed in a study involving 11 patients (121). All participants achieved at least a 5% BMI reduction, with 81% achieving reductions exceeding 10%, alongside decreased hunger ratings. More recently, results of the prospective, randomized, placebo-controlled TRANSCEND trial (NCT05774756), which enrolled 120 individuals with acquired hypothalamic obesity, have provided further support for the therapeutic potential of setmelanotide (122, 123). After 52 weeks of treatment, participants receiving setmelanotide (n=81) exhibited a mean BMI reduction of 16.5% (p<0.0001), whereas those assigned to placebo (n=39) showed a mean increase in BMI of 3.3%. Additionally, 80% of treated individuals achieved a reduction in BMI of at least 5% by week 52. In participants aged ≥12 years, the reduction in weekly average maximal daily hunger score was significantly greater in the setmelanotide group compared with placebo (−2.73 *vs*. −1.45; P = 0.009). In patients aged ≥4 years, hunger and hyperphagia were significantly reduced. Collectively, these findings suggest that setmelanotide may for the first time represent an effective treatment option for acquired hypothalamic obesity (124). As of March 2026, setmelanotide is now approved in the US to reduce excess body weight and maintain reduction long-term in adults and pediatric patients aged 4 years and older with acquired hypothalamic obesity (125).

Mechanistically, setmelanotide exerts its effects through activation of MC4R within the hypothalamus, thereby restoring disrupted satiety signaling pathways and promoting weight loss. However, given that hypothalamic injury is a defining feature of acquired hypothalamic obesity, the extent to which this mechanism remains functional requires careful consideration. In many cases, hypothalamic damage is incomplete, allowing for preservation of residual neuronal activity. Under such conditions, pharmacological activation of MC4R may stimulate remaining hypothalamic circuits and partially re-establish appetite regulation. Supporting this concept, Siljee et al. demonstrated that MC4R mRNA is widely expressed throughout the human hypothalamus, with notable co-localization alongside key regulatory neuropeptides, including alpha-melanocyte-stimulating hormone and agouti-related peptide (126).

#### 3.3.3 Bariatric interventions

Five studies explored the relationship between bariatric treatments and eating behavior (90, 127–130). A cross-sectional study found that individuals with hypothalamic obesity, including those with craniopharyngioma, experienced less weight loss following bariatric surgery compared to controls without tumors (90). Measures of satiety were also lower, though not statistically significant. Gastric bypass procedures were associated with sustained weight loss (131–133) and reduced hunger or food cravings over periods of 1.5–3 years (127, 129, 134), alongside modest increases in cognitive dietary restraint (129). Gastric banding was linked to BMI stabilization and reduced food-related addictive behaviors (128).

#### 3.3.4 Deep brain stimulation

Deep brain stimulation (DBS) has emerged as an innovative neuro modulatory approach with potential therapeutic value in management of hypothalamic obesity following craniopharyngioma (135, 136). DBS delivers high-frequency electrical stimulation to specific neural targets, thereby producing a functional modulation of pathological brain circuits. This technique has already demonstrated clinical efficacy in neurological and psychiatric disorders such as Parkinson disease, epilepsy, dystonia, and obsessive–compulsive disorder. Early applications in eating disorders (137), including anorexia nervosa and obesity, have also yielded encouraging findings. To date, three studies have evaluated DBS for hypothalamic obesity in six patients, including five individuals with Prader–Willi syndrome and one patient with craniopharyngioma. Stimulation targets included the nucleus accumbens and the lateral hypothalamic area. In the reported patient with craniopharyngioma, DBS targeting the nucleus accumbens was associated with a 8.7% BMI reduction (138). Evidence regarding long-term efficacy, durability of response, and safety remains unavailable.

#### 3.3.5 Truncal vagotomy

Truncal vagotomy, either as a standalone intervention or combined with bariatric procedures, has also been proposed as a potential treatment for hypothalamic obesity. The rationale is based on reducing vagal efferent signaling to pancreatic β-cells and adipose tissue, thereby influencing metabolic regulation (139). Lustig reported the use of laparoscopic truncal vagotomy in four individuals with hypothalamic obesity, with early outcomes suggesting potential benefit and relatively few complications or adverse effects (139). However, long-term data regarding sustained efficacy, safety, and tolerability are currently lacking.

### 3.4 Endocrine responses

Four case–control studies examined associations between eating behavior and entero-endocrine responses (86, 140–142). Findings suggest that altered hormonal responses, including GLP-1, ghrelin, PYY, and oxytocin, may contribute to dysregulated eating behavior. For example, reduced postprandial oxytocin levels were associated with increased concerns about eating and weight (86), while elevated GLP-1 levels and hyperphagia scores were observed in individuals with hypothalamic obesity (140). Additional studies reported altered ghrelin dynamics and inconsistent associations with hunger perception and satiety (69, 93, 141).

### 3.5 Autonomic nervous system

Lustig et al. (139) proposed that a loss of hypothalamic inhibitory control leads to increased vagal efferent activity, resulting in excessive stimulation of pancreatic β-cells. This hyperstimulation promotes hyperinsulinemia and thereby contributes to the development of severe obesity. Based on this concept, they evaluated the somatostatin analogue octreotide, which suppresses β-cell activity (143). However, its clinical utility has been limited by modest efficacy and a high rate of adverse effects, including gallstones and gastrointestinal complaints. Additional investigations (144) have suggested that diminished central sympathetic nervous system activity may contribute to the reduced physical activity and severe obesity observed in these patients. In support of this hypothesis, Roth et al. (145) demonstrated decreased urinary concentrations of catecholamine metabolites, which were associated with obesity and lower levels of physical activity.

### 3.6 Energy expenditure

Several studies reported reduced resting energy expenditure or basal metabolic rate in individuals with craniopharyngioma (62, 65, 146), with some also noting reduced energy intake (63, 65). However, other studies found no significant differences compared to controls (82, 87). Exenatide treatment was associated with reduced energy intake and total energy expenditure in one RCT (107), while another study reported decreased caloric intake and variable weight outcomes without changes in resting energy expenditure (84).

### 3.7 Neuropsychology

Limited evidence exists regarding neuropsychological correlates of eating behavior (21, 76, 147, 148). One study demonstrated associations between tumor size, weight gain, and hyperphagia (149), while another reported behavioral disturbance related to excessive food-seeking in children (148).

### 3.8 Neuroradiological imaging

Functional imaging studies indicate altered neural responses to food-related stimuli in individuals with hypothalamic damage, including reduced activation in reward-related brain regions and increased responsiveness to high-calorie food cues (90, 150). Structural imaging findings suggest that greater hypothalamic involvement is associated with more severe eating disturbances and obesity (86, 151). Yurddas et al. (152) observed that high hypothalamic post-operative T2 SI, suggestive of inflammation or edema, correlated with BMI increase and presence of post-surgical hypothalamic obesity and was linked to older age, less cystic tumors, and higher Müller grade of hypothalamic damage.

## 4 Summary and discussion

### 4.1 Main findings

This review identified 82 studies investigating eating behavior in patients with craniopharyngioma. However, only one study compared eating behavior between patients with and without obesity (83), and only one evaluated a pharmacological intervention specifically targeting eating behavior and hunger (123). Based on the studies reviewed, it appears that several interventions may improve BMI or weight-related outcomes, but have limited effects on eating behavior itself. In contrast, setmelanotide appears to have a more direct impact on hyperphagia and eating behavior (123), which may be particularly relevant given its recent approval for hypothalamic obesity. No studies compared childhood-onset with adulthood-onset craniopharyngioma. Overall, the evidence base was limited by low methodological quality, reflecting the challenges of studying a rare, heterogeneous disorder with multifactorial contributors to obesity.

### 4.2 Eating behavior in craniopharyngioma

Although eating behavior is likely to contribute substantially to weight gain, evidence for pathological eating patterns remains limited. Available studies suggest increased hyperphagia, heightened hunger perception, impaired dietary restraint, and more frequent snacking between meals in affected patients (86, 87, 89, 117), 153). Only one study reported greater pathological eating behavior among patients with obesity than those without (83). Pharmacological interventions represented the largest research focus, with eight studies demonstrating reductions in hunger or hyperphagic symptoms following treatment (98, 101, 103, 104, 108, 109, 118). Evidence from three studies further suggests that tumor location may be associated with adverse eating behaviors, indicating potential value for early risk stratification at diagnosis (75, 86, 151).

### 4.3 Quality of evidence

The rarity of craniopharyngioma has resulted in predominantly small studies, with the largest including 101 patients with childhood-onset disease (83). Most publications were case reports or case series, limiting statistical power despite frequent matching for age, sex, and BMI. Furthermore, validated measures of eating behavior were used infrequently, with many studies relying on non-standardized instruments. Although patient-reported outcomes are essential for assessing eating behavior, they remain susceptible to recall bias (154). Long-term evidence, particularly for pharmacological therapies, is also scarce, although one randomized controlled trial reported favorable effects of setmelanotide on hunger and BMI (123).

### 4.4 Measuring eating behavior

Only seven studies assessed eating behavior as a primary outcome; most considered it as a secondary endpoint or described it anecdotally. Among studies using questionnaires, 20 studies (63, 81, 83, 86, 87, 89–91, 105, 107, 110, 111, 140, 141, 150, 155, 156), 123) employed validated instruments (157–175) (**Table 1A**). Future studies should prioritize standardized tools, including the TFEQ (157), C/AEBQ (158), and DYQ (159). Considerable clinical heterogeneity with regard to tumor location and size, treatment modality, and obesity severity, likely contributes to variability in eating behavior. As the underlying mechanisms remain poorly understood, further research is required to identify determinants of obesity susceptibility in patients with craniopharyngioma.

### 4.5 Strengths and limitations of the review

A comprehensive search strategy was employed to maximize study identification. The review adhered to established methodological guidance for systematic narrative reviews, including rigorous screening, data extraction, and reporting procedures (176). Nevertheless, the conclusions are constrained by the limited quality, heterogeneity and low level of available evidence. Furthermore, most studies are focusing on short-term weight development, whereas long-term assessment of weight development, potential adverse effects of medication, and the focus on underlying eating behavior are scarce.

### 4.6 Recommendations for future research

Prospective, multicenter studies using validated assessments of eating behavior are needed to improve risk stratification and evaluate multimodal treatment strategies. Future trials should investigate integrated approaches combining optimized pituitary hormone replacement with pharmacological therapies for hypothalamic obesity, including MC4R agonists together with GLP-1 receptor agonists and/or stimulant medications (177–179). Such strategies may improve long-term metabolic outcomes and quality of life. Evidence from psychological research further suggests that behavioral interventions targeting responsiveness to energy-dense foods can reduce energy intake and obesity over time (180). Combining these interventions with effective pharmacological therapies, such as setmelanotide, may be particularly beneficial for patients with severe hyperphagia and obesity.

### 4.7 Recommendations for clinical practice

Disordered eating behavior and hypothalamic obesity are major complications of craniopharyngioma. Pharmacological treatments, particularly GLP-1 receptor agonists and MC4R agonists such as setmelanotide, have shown encouraging efficacy, with the latter demonstrating particularly promising results (123). These agents are effective in both pediatric and adult obesity (123, 181) and appear beneficial in patients with craniopharyngioma despite structural hypothalamic damage caused by the tumor or its treatment (84, 107). However, long-term efficacy and adverse effects are not sufficient characterized. Routine longitudinal assessment using standardized, validated measures may facilitate early identification of hyperphagia and disordered eating, enabling timely preventive interventions before obesity becomes established.

## 5 Conclusions

High-quality evidence examining eating behavior as either a primary outcome or therapeutic target in craniopharyngioma remains limited. Well-designed multicenter studies are needed to clarify the mechanisms underlying excessive weight gain, identify patients at greatest risk, and establish effective interventions. In light of promising RCT findings (122, 123), there is a clear need to establish novel personalized treatment algorithms for hypothalamic obesity. Future patient cohorts with acquired hypothalamic obesity should be managed by experienced multidisciplinary teams using standardized diagnostic pathways, systematic risk stratification, and structured prospective assessment of multimodal therapeutic strategies. Such approaches may include optimized pituitary hormone replacement alongside combination pharmacotherapy targeting hypothalamic obesity with MC4R agonists used in conjunction with GLP-1 receptor agonists and/or stimulant agents. The long-term efficacy and potential adverse effects of such combination therapies still have to analyzed. The development of individualized treatment frameworks has the potential to deliver urgently needed improvements in metabolic health, clinical outcomes, and quality of life for this underserved population affected by disordered eating and severe obesity. Furthermore, improved understanding of eating behavior may inform such targeted therapies and provide a valuable outcome measure for evaluating treatments aimed at reducing obesity-related complications and enhancing quality of life following craniopharyngioma treatment.
